# An Infoveillance System for Detecting and Tracking Relevant Topics From Italian Tweets During the COVID-19 Event

**DOI:** 10.1109/ACCESS.2020.3010033

**Published:** 2020-07-17

**Authors:** Enrico De Santis, Alessio Martino, Antonello Rizzi

**Affiliations:** Department of Information Engineering, Electronics and TelecommunicationsUniversity of Rome “La Sapienza,” 00184 Rome Italy

**Keywords:** Natural language processing, topic tracking, topic detection, social network analysis, text mining, COVID-19, infodemiology, infoveillance

## Abstract

The year 2020 opened with a dramatic epidemic caused by a new species of coronavirus that soon has been declared a pandemic by the WHO due to the high number of deaths and the critical mass of worldwide hospitalized patients, of order of millions. The COVID-19 pandemic has forced the governments of hundreds of countries to apply several heavy restrictions in the citizens’ socio-economic life. Italy was one of the most affected countries with long-term restrictions, impacting the socio-economic tissue. During this lockdown period, people got informed mostly on Online Social Media, where a heated debate followed all main ongoing events. In this scenario, the following study presents an in-depth analysis of the main emergent topics discussed during the lockdown phase within the Italian Twitter community. The analysis has been conducted through a general purpose methodological framework, grounded on a biological metaphor and on a chain of NLP and graph analysis techniques, in charge of detecting and tracking emerging topics in Online Social Media, e.g. streams of Twitter data. A term-frequency analysis in subsequent time slots is pipelined with nutrition and energy metrics for computing hot terms by also exploiting the tweets quality information, such as the social influence of the users. Finally, a co-occurrence analysis is adopted for building a topic graph where emerging topics are suitably selected. We demonstrate via a careful parameter setting the effectiveness of the topic tracking system, tailored to the current Twitter standard API restrictions, in capturing the main sociopolitical events that occurred during this dramatic phase.

## I. Introduction

It is now well established that Internet and, in particular Online Social Media (OSM), are an invaluable source of *fresh* information. OSM have been widely adopted as means of news dissemination, event reporting, opinion expression and discussion [1]. Since 2006, the American online microblogging platform and social network service Twitter has gained rapidly more and more worldwide popularity with 321M active users in 2019. Twitter online operations started as a very short text message service provided by users via SMS or online platform. Currently, after a rapid and continuous evolution both from the technical point of view and in the diverse segments of the population reached worldwide, it is an affirmed OSM conceived as a mixture of news media and social network features. Considering the mass of active users and how they interact with the platform – many of them can be considered as sensors or amplifier of facts or happening events – the Twitter data stream possess an invaluable strength in the task of discovering and tracking real-world events. In fact, a vast literature shows how the Twitter data stream can be used for discovering, tracking and analyzing these real-world events, such as earthquakes and natural disasters [2]–[4] in earth science, or national security events such as terrorists attacks [5]–[7]. Furthermore, Twitter data have been widely used even for tracking and analyzing important sociopolitical events, such as the riots during the Arab Spring [8] and the process of opinion formation around major political themes [9]–[12], with particular attention to disinformation spreading [13].

Interestingly, Twitter has been used even for Public Health Monitoring tasks [14], specifically during pandemic crisis such as the influenza A H1N1 or swine flu in 2009 [15], [16]. Hence, OSM can be nowadays fruitfully used to study the dynamics of real-world events and monitoring such phenomena can have a direct implication on the possibility of understanding and describing their evolution, aiming to better decision making procedures for political decision makers and democratic institutions. In particular, a tracking system able to sense the Twitter stream to leverage fresh information in terms of emerging topics can be useful for early-detecting anomalous activities, preventing possible misuses of the OSM.

In this paper it is faced the analysis problem of the Italian Twitter community through a suitable topic tracking methodology during the lockdown period in Italy, subsequent to the dramatic COVID-19 pandemic. At the time of writing, the COVID-19 pandemic – also known as the *coronavirus* pandemic – is an ongoing pandemic of coronavirus disease in 2019 (hence COVID-19). It is caused by severe acute respiratory syndrome coronavirus 2 (SARS-CoV-2) and the outbreak was first identified in Wuhan, mainland China, in December 2019 [17]. The World Health Organization (WHO) declared the outbreak a pandemic on 11 March 2020 and, as of June of the same year, more than 8.4 million cases of COVID-19 have been reported in more than 188 countries, resulting in more than 450,000 deaths with more than 4.1 million people that have been recovered worldwide.^1^ In Italy, on 4 March 2020, after the detection of the first 100 death related to the pandemic, the government has ordered the complete closure of all schools and universities of all levels. On 11 March 2020, Italian Prime Minister Giuseppe Conte ordered a set of severe confinement measures and the so-called *social distancing*, together with the interruption of numerous productive, commercial and professional activities. Hence, the pandemic generated a worldwide dramatic situation never seen before with repercussions even on the economic scenario and, during the period that spans from March to June, the Italian population was constrained at home for safety reasons, acquiring important information mostly on social network platforms. The insane information flow about the pandemic enriched with fake-news has declared by WHO as a serious *infodemic* problem [18]–[20]. Eysenbach stated in early 2000 that infodemiology is a new research discipline and methodology related to the study of the determinants and distribution of health information and misinformation which may be useful in guiding health professionals and patients to quality health information on the Internet [18]. The WHO Director-General Tedros Adhanom Ghebreyesus at the Munich Security Conference on 15 February 2020 declared [21] “We’re not just fighting an epidemic; we’re fighting an infodemic”. This mean that the risk of *false information* [22] (i.e. forms of falsehood, including rumors, hoaxes, myths, conspiracy theories and other misleading or inaccurate) is very high. Covid-19 is a phenomenon of enormous magnitude and relevance with a great impact on the media system [23]. With the starting of COVID-19 pandemic, we are assisting to a growing number of infodemiology studies [24]–[27] where, interestingly, the spread of news or rumors are evaluated with the same epidemic models adopted in real-world epidemics [28], for example measuring a $R_{0}$ parameter that, if found higher than the unitary value, it announces an infodemic. In light of an infoveillance study over the English speakers’ Twitter community, authors in [29] analyze 167073 tweets, collected from the beginning of February 2020 to mid-March 2020, through word frequencies and the Latent Dirichlet Allocation (LDA) approach, aiming to identify the most common topics in the tweets. The analysis identifies 12 topics, which were grouped into four main themes: origin of the virus; its sources; its impact on people, countries, and the economy; and ways of mitigating the risk of infection. As expected, the impact on people and the economy is not to be underestimated. However, the methodologies adopted in infoveillance and infodemiology studies differ in the specific goals of the analysis, in the data sources and in the approaches, which span from correlation assessments to advanced machine learning systems. In this universe, it is important having available a system able to promptly trigger facts and events online. Moreover, in this study, we adopt an extended meaning of the term “infoveillance” compared to the traditional one [19], in that the COVID-19 pandemic impacts not only on public health debate but even in every social and economical facet, transforming safety issues in public security issues.^1^COVID-19 Dashboard by the Center for Systems Science and Engineering (CSSE) at Johns Hopkins University (JHU), https://gisanddata.maps.arcgis.com/apps/opsdashboard/index.html#/bda7594740fd40299423467b48e9ecf6

The following analysis focuses precisely on the early period of COVID-19 pandemic, during which a large dataset of tweets (in Italian language) has been collected through the Twitter Streaming APIs. The main aim of this work is to track the emergent topics within the general debate in Italy during the pandemic. For this purpose, a topic tracking system is constructed grounding on the methodological framework presented in [30], adapting the main functions both to the deep change in Twitter APIs (for example, on the restriction of available data and the increasing in length of text messages) and to the current case study. The methodology allows tracking emerging topics grounding on monitoring emerging terms by adopting a series of Natural Language Processing and graph-based techniques. A *topic* is defined as a coherent set of semantically related terms that express a single argument. *Hot terms* are term heavily used during a long time period, while a term is emergent if it results to be hot in the considered time interval but not in the previous ones. Interestingly, the methodology is mediated by a biological metaphor, where the life-cycle of a keyword (word) can be considered as analogous to the one of a living being. Specifically, within a Content Aging Theory framework [31], a keyword is like a biological system that, if it is fed by a well-suited amount of *nourishment*, then its life-cycle is prolonged, while as soon as it is no longer available the living organism likely dies. The nourishment for a keyword is provided by its occurrence statistics in a set of tweets in a time interval – measured through a Term Frequency (TF) term – and the *quality* of tweets (containing the given keyword), measured by a *social influence* value related to the user that generated the contents. In this study, the nourishment term is further increased if the given keyword is even marked as a hashtag, with the aim of providing more semantic strength to the considered keyword that can be, in this way, a bearer of meaning. The tracking and the detection of emergent terms and topics are obtained considering a sequence of time intervals in which is measured the vitality of the keyword through an *energy* quantity that takes into account both the difference in the nutrition term in different time intervals and the amount of time flow. The energy quantities and a co-occurrence analysis in different time windows allow building a graph containing emerging keywords and common words. Through a suitable algorithm, a partition of the co-occurrence graph is further obtained where sub-graphs are conceived as emergent topics for the given time interval.

This paper is organized as follows: in Section II the related works are revised, while in Section III the methodological framework is resumed. In Section IV the results of the analysis are presented and discussed. Conclusions are drawn in Section V. Finally, in Appendix, a glossary of main Italian terms, people and abbreviations is provided.

## II. Related Works

Topic Detection and Tracking aims at the extraction of topics from a collection (or stream) of texts in order to study and quantify their importance (“trend”) over time [32]. As aptly discussed in [33], there are two main families of techniques in order to perform topic detection: document-pivot and feature-pivot. The main difference is that, in the former case, documents are clustered together, whereas in the latter case keywords or individual terms are clustered together.

That said, within the document-pivot family, research works such as [34]–[36] leveraged on Term Frequency-Inverse Document Frequency (TF-IDF) in order to map documents towards a suitable vector space [37]. On occasion, other features can be considered alongside TF-IDF, such as time proximity between tweets [38].

Feature-pivot methods, as instead, heavily rely on statistical topic models, with the final goal of extracting ‘hot terms’ that describe a given topic. Within this family, LDA [39] plays a huge role [40]–[43]. Other techniques include the study of the *burstiness* of given terms, with the rationale that ‘hot topics’ spread rapidly on social media as soon as they are first announced [44]–[47]. An alternative approach, pursued in this work, is the use of graphs in order to capture the co-occurrences of terms: in fact, a graph is able to encode the pairwise similarities between nodes, which can either be single terms [30], [48], [49] or short sentences [50]. This allows to cast the topic detection problem into a community detection problem defined on a graph.

The vast majority of the aforementioned works deals with ‘topic detection’. However, as discussed in [40] ‘topic detection’ is just one of the two building blocks in Topic Detection and Tracking, the other being indeed ‘topic tracking’. Topic tracking can also be performed according to different strategies, including clustering [51], online variants of LDA [40], [52] or by exploiting and studying temporal dynamics over a pre-defined time window [30], [53].

The work by [30] serves as a starting point for this paper. Their work can be summarized as a five-steps procedure which starts by collecting tweets, then computing the energy of the terms by considering a given time window, selecting emerging terms according to their energies and building a co-occurrence graph amongst emerging terms. Finally, topics are collected from the resulting graph. In this paper, we perform some modification of the original pipeline proposed in [30] in order to address updates and changes in the Twitter API and in order to better suit our case study, that is, topic detection and tracking on COVID-19-related tweets: this period, although dramatic, represents a more unique than rare opportunity for this kind of work. Hence, we collected tweets everyday for about three months during the lockdown phase in Italy.

## III. Methodology

### A. Data Collection

For the current study, we built a dataset of 1044645 tweets through a suitable listener connected to the standard Twitter Streaming API, accessible with a Twitter developer account. The Twitter Streaming API works like a radio receiver tuned on a specific radiofrequency that captures on-air programs in real-time. In fact, the Streaming API allows capturing streaming Twitter content selecting a set of keywords. The listener object has been set to collect a stream filtering for a time period that spans from 9 March to 5 June 2020, for the following Italian keywords: Salvini, Conte, PD, salvini, conte, pd, lega, Lega, coronavirus, Coronavirus, calcio, Calcio, sport, Sport, UE, ue, europa, Europa, USA, NBA, carceri, carcere, virus, meloni, Meloni, coni, CONI, renzi, Renzi, borsa, Borsa, Trump, NASA, ESA, scienza. The semantic of the selected 35 keywords have been chosen with the aim of offering a wide coverage of the main buzzing topics not focusing only on the COVID pandemic, but also to a more general socio-political scenario. In fact, maybe for the first time, a worldwide pandemic meets a globalized and interconnected world and issues overcome the public health safety invalidating the socio-economic tissue. For example, the tightness of the European Union has been severely put under pressure by the pandemic. Hence, both from a infoveillance and security viewpoint the selected keywords – see the glossary in the Appendix for a deeper explanation – cover the COVID-19 pandemic along with the internal and external economic and political scenario, the general scientific debate and sports. Tweets are filtered for the Italian language (‘it’) exploiting the specific filtering function available in the Twitter Streaming API. All collected tweets have been separated on a daily basis with an average of 20000 tweets per day.

### B. Data Preprocessing

A marked difference with the original methodology proposed in [30] is in the adoption, in the current study, of several preprocessing steps. The motivation is two-fold. With no preprocessing, the final outputs are noisy and the computational time of the entire algorithm pipeline is obviously higher due to such noise. The adopted preprocessing steps are the following:

• text tokenization with the aid of Part-of-Speech information;
• hashtags extraction;
• lower casing conversion;
• links, symbols, emojis and retweets removals;
• stop-words removals (Italian words most commonly used stored as a list in an external file);
• text lemmatization (optional): similar to stemming, associates to every word its lemma;
• numbers removals (optional).

The topic tracking system is designed in a versatile fashion, hence some preprocessing steps are optional and leaved as a choice to the end-user. The lemmatization step, whether selected, is performed with the TreeTagger wrapper [54], [55].

### C. Topic Detection and Tracking

The main aim of the topic tracking system is tracking emerging topics on the Twitter Italian community in a given time interval. Hence, within a time interval $r$ set by the user, the $t$-th time interval $I^{t}$ is defined as:

\begin{equation*} I^{t}=\left \langle{ i_{t},i_{t}+r}\right \rangle \tag{1}\end{equation*}

where $i_{t}$ is the starting instant of the $t$-th considered time interval (the value 0 is the first instant). For each time interval $I^{t}$ a corpus of $n$ tweets $\left |{TW^{t}}\right | $ is collected and to each tweet $j$ it is associated a suitable vectors of weights $\mathbf {tw}_{j}=\left [{w_{j,1}, w_{j,2}, \ldots,w_{j,v}}\right]$ where $v$ is the cardinality of the keywords vocabulary $K^{t}$.

The weight $w_{j,x}$ for the $x$-th vocabulary term and for the $j$-th tweet is given by the augmented term frequency [56]:

\begin{equation*} w_{j,x}=0.5+0.5 \cdot \frac {tf_{j,x}}{tf_{j}^{\text {max}}}, \tag{2}\end{equation*}

where $tf_{j,x}$ is is the term frequency value of the $x$-th vocabulary term for the $j$-th tweet and $tf_{j}^{\text {max}}$ is the highest term frequency value of the $j$-th tweet. Hence, for each time interval, each tweet is represented as a weight vector that resumes the statistical information related to each pertaining term.

In order to compute the *hot terms* in a given time interval and the main topics in a suitable way, it is important to define two main concepts, that are the *content nutrition* and *content energy*. It is possible to imagine that each tweet provides its own keywords by a quantity called *nutrition* whose quality is given by the authority of the user that produced the tweet. In this way, different tweets containing the same keywords can receive different nutrition values depending on the representativeness of the user that produced the tweets. With difference to [30], in this study the quality of the nutrition is given even considering if the keyword is used as hashtag.

Hence, considering a keyword $k \in k^{t}$ and the set of tweets $TW^{t}_{k} \in TW^{t}$ containing a term $k$ at time interval $I^{t}$, the amount of nutrition for a keyword $k$ is defined as:

\begin{equation*} \text {nutr}_{k}^{t}=\sum _{tw_{j} \in TW_{k}^{t}} h \cdot w_{k,j} \cdot \text {auth}(\text {user}(\mathbf {tw}_{j})), \tag{3}\end{equation*}

where $w_{k,j}$ is the weight of the keyword $k$ for the tweet $j$ (in the tweet vector $\mathbf {tw}_{j}$), $h$ is a constant that boosts the nutrition if the keyword is also an hashtag, and $\text {auth}(\text {user}(\mathbf {tw}_{j}))$ is a numerical value indicating the representativeness of the tweet author.

There are a number of methods for measuring the importance of a source in terms of several features related to the social influence of a user [57]. In their original work [30] adopt an authority graph and the PageRank algorithm [58] to estimate the social influence. They state that a Twitter user can follow the text stream of other users by expliciting the social relationship of follower. On the other hand, a user who is being followed by another user does not necessarily have to reciprocate the relationship by following it back, which makes the graph of the network directed. By the way, the Twitter public Streaming APIs make available only a subset of information about the author of a tweet and in this subset is unavailable the follower-followee list for build the social graph. Moreover, the computation of such a graph can be quite expensive. Thus, in the current study, we adopt a simple formulation – both from the computational point of view and exploiting the current available information about tweets’ authors – of the social influence of a user $u_{i}$ through the number of followers and followees:

\begin{equation*} \text {auth}(u_{i})=\frac {\text {followers}(u_{i})}{\text {followers}(u_{i})+\text {followee}(u_{i})}. \tag{4}\end{equation*}

Finally, for each keyword $k$ adopted in the Twitter community in a time interval $I^{t}$, the nutrition amount evaluates the usage of this term by considering i) its frequency appearance in tweets, ii) the social influence of the source that reports the keyword $k$, iii) the possibility that the keywords has a strong semantic content (in the specific time interval) being an hashtag. Hence, the topic tracking system is in charge of evaluating the frequency of key terms and their relevance qualified by the user authority and the particular meaning in the specific contest.

The nutrition for a keyword helps to defining another important quantity that is the the *energy* of a term. The energy is related to effective contribution, that is how much a term is emergent, in the corpus of tweets. The energy is the key value to compute the set of *hot terms*, where ‘hotness’ is related to the extensiveness of the usage within the considered time interval. The energy helps also to compute the emergence of a term, where a keyword is ‘emergent’ if it results to be *hot* in the considered time interval but not in the previous ones [30]. By these definitions, a hot term is different from an emergent term. It is possible to have a hot term (heavily used) that is not emergent in a time interval because the usage is quite constant in it.

The energy is computed considering a parameter $s$ ($0 < s < t$), that limits the number of previous time slots considered to analyze the keywords life cycles, hence defining the history worthiness of the resulting emerging keywords. Given a keyword $k$, the energy value in a time interval $I^{t}$ is:

\begin{equation*} \text {energy}_{k}^{t} = \sum _{x=t-s}^{t} ((\text {nutr}_{k}^{t})^{2} - (\text {nutr}_{k}^{x})^{2}) \cdot \frac {1}{t-x}, \tag{5}\end{equation*}

where $\text {nutr}_{k}^{x}$ represents the nutrition obtained by the keyword $k$ during the interval time $I^{x}$. It is worth to note that Eq. (5) allows quantifying the usage of a given term with respect to its previous usages in a limited number of time intervals. It takes into account i) the difference in terms of usage of a given keyword by considering the difference of nutritions received in the time frames $I^{x}$ and $I^{t}$ ($x < t$), ii) the temporal distance among the two considered intervals.

The *hot* and the *emergent keywords*, within this framework, allows computing the *emergent topics*. It is important first defining a set of emerging terms through a critical drop value represented by a user-defined threshold $\delta \geq 1$:

\begin{equation*} \text {drop}^{t}= \delta \cdot \frac {\sum _{k \in K^{t}}(\text {energy}_{k}^{t})}{\left |{K^{t}}\right |}. \tag{6}\end{equation*}

By using Eq. (6) it is possible to define the set of emerging keywords $EK^{t}$ as:

\begin{equation*} \forall k \in K^{t}, k \in EK^{t} \iff \text {energy}_{k}^{t} > \text {drop}^{t}. \tag{7}\end{equation*}

Hence, the parameter $\delta $ rules the number of extracted hot terms. We remark that authors in [30] suggest that it is possible to compute the set of emergent terms even in an unsupervised fashion, without setting a threshold parameter. In this study, we refer to the supervised way, that is adopting a user-defined threshold, since this method is found more reliable, as reported even by the authors themselves.

To finally reach the definition of emerging topics – related to the emerging keywords – the system needs to analyze the semantic relationships of keywords through the co-occurrence information in the considered whole time interval. Hence, it is possible to define a correlation vector $\textbf {cv}_{k}$ to each keyword $k \in K^{t}$. The correlation vector captures the relationships among the keyword $k$ and all others terms in the given time interval. This is done by computing the degree of correlation between keywords $k$ and $z$ by using the set of tweets containing both terms as positive evidence of the relatedness of the two terms. On the contrary, the set of tweets containing only one of them represents a negative evidence. This idea is captured by the following formula that represent a probabilistic feedback mechanism [59]:

\begin{align*} cc_{k,z}^{t}=&\log \frac {r_{k,z}/(R_{k}-r_{k,z})}{(n_{x}-r_{k,z})/(N-n_{z}-R_{k}+r_{k,z})} \cdot \\&\cdot \,\left |{\frac {r_{k,z}}{R_{k}} - \frac {n_{z} - r_{k,z}}{N-R_{k}}}\right |,\tag{8}\end{align*}

where:

• $r_{k,z}$ is the number of tweets in the interval containing both keywords $k$ and $z$;
• $n_{z}$ is the number of tweets containing the keyword $z$;
• $R_{k}$ is the number of tweets containing $k$;
• $N$ is the total number of tweets.

Hence, a given term $k$ is associated to a correlation vector:

\begin{equation*} \mathbf {cv}_{k}^{t}=\left \langle{ c_{k,1}, c_{k,2}, \ldots,c_{k,v} }\right \rangle, \tag{9}\end{equation*}

where $v=\left |{K^{t}}\right |$. The elements $c_{k,i}$ represent the correlation between the term $k$ and the term $i \in K^{t}$ at the time interval $I^{t}$.

At this point, the correlation vector $\textbf {cv}_{k}^{t}$ can be used for identifying the main emerging topics related to emerging terms retrieved during the given time interval. Specifically, a directed keyword-based topic graph $TG^{t}(K^{t},E,\rho)$, can be constructed. $K^{t}$ is the set of vertices of which the elements are the keywords $k \in K^{t}$ retrieved during the time interval $I^{t}$. Given two keywords $k,z \in K^{t}$ such that $\mathbf {cv}_{k}^{t}\left [{z}\right]\neq 0$, there exists an edge $\left \langle{ k,z}\right \rangle \in E$, such that:

\begin{equation*} \rho _{k,z}=\rho (\left \langle{ k,z}\right \rangle)=\frac {\mathbf {cv}_{k}^{t}\left [{z}\right]}{\left \|{\mathbf {cv}_{k}^{t}}\right \|}. \tag{10}\end{equation*}

In the above Eq. (10), $\rho _{k,z}$ is the relative weight of the keyword $k$ in $\mathbf {cv}_{k}^{t}$, that is the role of the keyword $z$ in the *context* of keyword $k$. In the current study the graph $TG^{t}(K^{t},E,\rho)$ is thinned by removing edges with values lower than a cutoff threshold $\phi $. This parameter is fundamental for the emerging topics retrieval in that a too small value results in a huge unique component, while a large value leads to a disconnected graph, making useless the below-described procedure for retrieving the topics.

The topological structure of the graph can be exploited for retrieving semantically-related keywords that are intended as an emerging topic. In particular, for each keyword $z \in EK^{t}$, an emerging topic is defined as the subgraph $ET_{z}^{t}(K_{z},E_{z},\rho)$ connecting keywords that are semantically related to the keyword $z$ within $I^{t}$. The subgraph is obtained as the set of vertices $S$ reachable from $z$ through a path computed by means of the Depth First Search algorithm. In other words, topics are represented by strongly connected components. Given the entire set of $n$ emerging keywords, $EK^{t}$ is computed as the corresponding set of emerging topics, namely the set $ET^{t}=\left \{{ET_{1}^{t}, ET_{2}^{t},\ldots,ET_{n}^{t}, }\right \}$ of strongly connected components. At the end of the procedure an emerging topic is represented by an emerging term $z$ and other semantically related common terms not necessarily included in $EK^{t}$, that can be thought popular terms (e.g. ‘Trump’). In a pictorial graph representation the connected components can be represented as colored vertices, while their dimension can represent if a term is an emerging term or not (an example will be provided in Section IV).

It is worth to note that the topic graph exploits the information leveraged from all tweets, even those that do not report emerging terms. Hence the current approach not only is able to retrieve such terms that directly co-occur with the emerging terms but we can also retrieve those which are indirectly related with the emerging ones. This is possible with term co-occurring with keywords that they themselves co-occur with the emerging terms.

Finally, to provide the user with insights of which topic is more important, topics can be ranked by considering the energies of the related emerging terms.

## IV. Empirical Results

Two different studies are performed in order to test the proposed approach. The first study aims at assessing the term energy evolution as function of time on a 30-days time horizon, whereas the second study aims at focusing on specific days in order to analyze their topics. The selection of terms (first analysis) and days (second analysis) is mainly driven by the events themselves: in fact, as clear from the previous section, the proposed system works in an unsupervised fashion. To this end, in order to check for the effectiveness of the approach, days with interesting events have been selected and validated a-posteriori. Same reasoning holds for the selection of terms for energy monitoring. As concerns the topics, several parameters are experimented, such as cutoff value $\phi $ for thinning the co-occurrence graph, the drop of value for retrieving emerging terms $\delta $, and the number of previous time windows to consider in the hot terms computing $s$. The ‘threshold’ parameter has been introduced to limit the number of words per topic. Finally, in the presented experiments, the lemmatization in the preprocessing step is not adopted.

### A. Monitoring Energy Evolution Through Time

**FIGURE 1.**
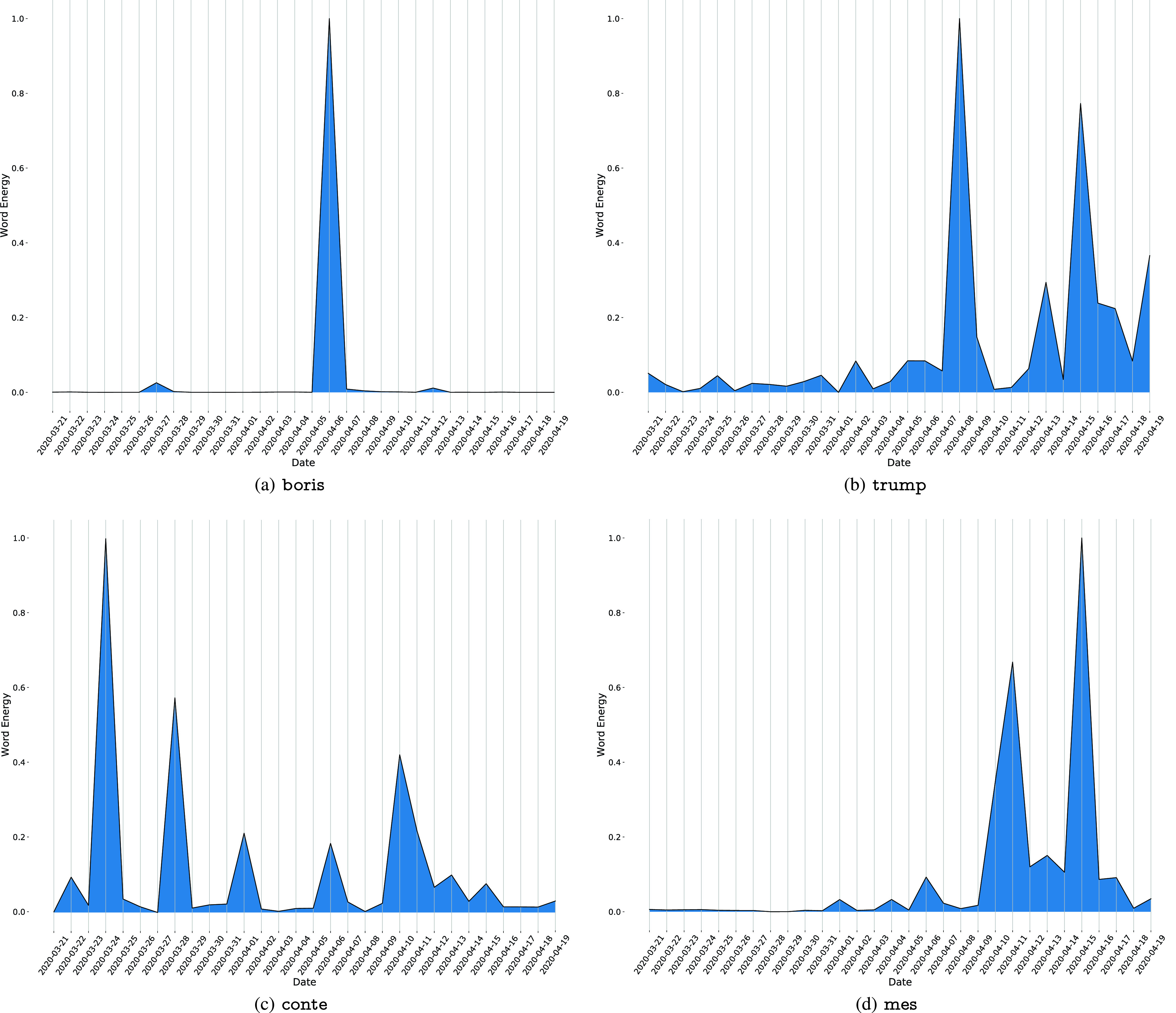
Term energies evolution through time. Energy values are normalized in range [0, 1].

In a first analysis, we show the energy evolution for some of the most relevant words in the considered time horizon. For example, Figure 1a shows the energy evolution for the word boris which sees a spike on 5 April 2020, the day in which he has been taken to hospital due to coronavirus.^2^ Similarly, Figure 1b regards the word trump, whose relevance on Twitter starts increasing from April, when the coronavirus pandemic started spreading in the U.S.A., and he started being a more common topic.^3^ Figure 1c shows the trend for the word conte, with spikes on 24 March 2020, 28 March 2020, 1 April 2020, 6 April 2020 and 10 April 2020: in these days Giuseppe Conte held press releases and interviews in order to discuss and introduce new rules and regulations during the lockdown phase in Italy.^4^ Finally, Figure 1d shows the energy evolution for the word mes, which became a hot topic in April due to the economic crisis due to the lockdown in Italy.^5^ We remark that the performances of the actual version of the topic tracking system, specifically in detecting buzzing topics, is satisfactory in that several buzzing keywords, for example related to the president Donald Trump, or even the president Giuseppe Conte are heavily and constantly used by a Twitter user, but only in a certain time, depending on underlying events, they are boosted and the system is in charge of detecting these events along with the related topics.^2^https://www.theguardian.com/politics/2020/apr/05/boris-johnson-admitted-to-hospital-with-coronavirus^3^https://abcnews.go.com/Health/coronavirus-map-tracking-spread-us-world/^4^http://www.governo.it/it/coronavirus-video (in Italian).^5^https://www.corriere.it/economia/risparmio/cards/cos-nuovo-mes-l-emergenza-coronavirus-ruolo-bce/mes-nuova-linea-credito-fronteggiare-coronavirus.shtml (in Italian).

### B. Daily Hot Topics

**FIGURE 2.**
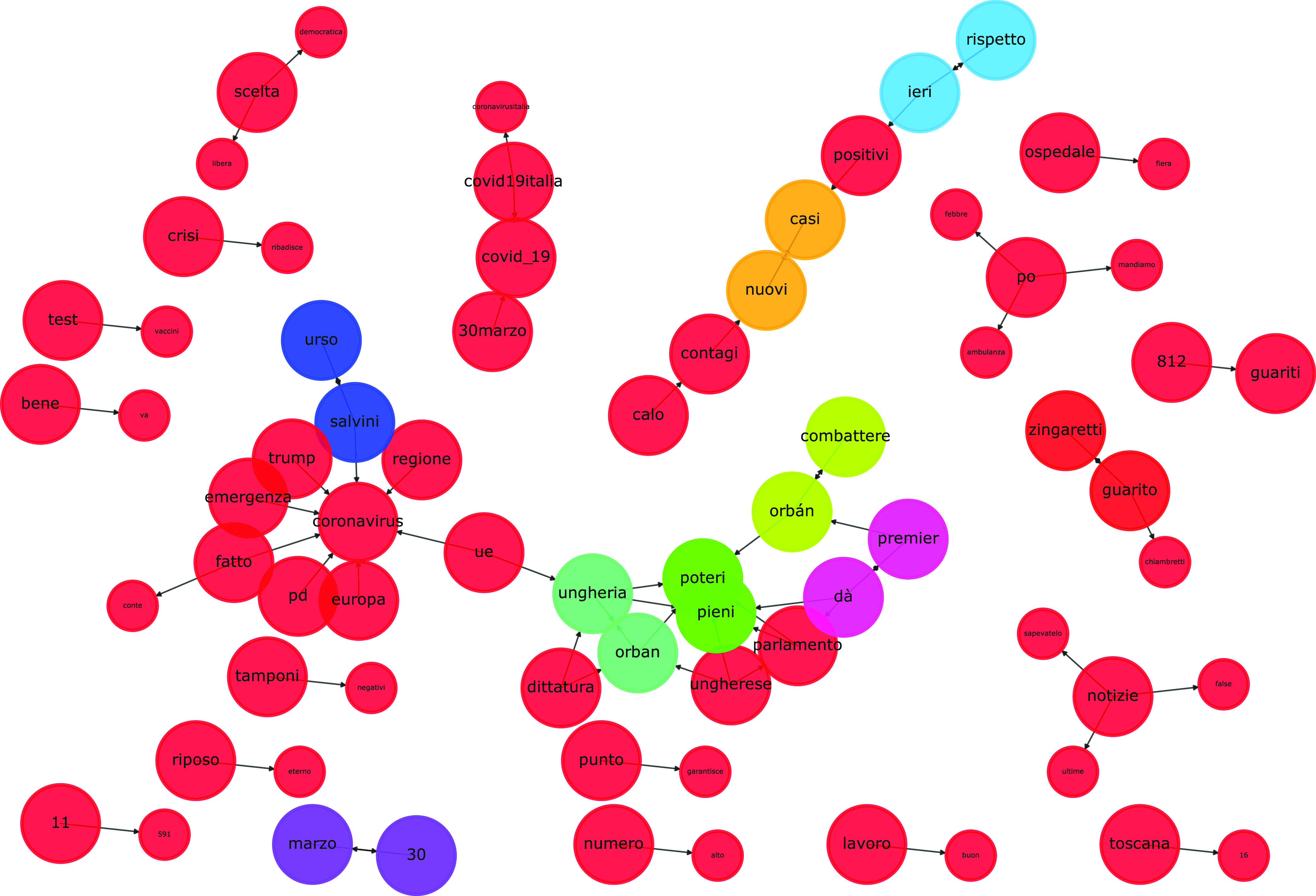
Graph-based topic representation (27 May 2020). No thresholding on the number of terms per topic.

In this second study, instead of focusing on the relevance of individual words over time, we focus analyzing topics on specific days within the considered time horizon. Topics are shown in Tables 1–7, with setup parameters reported in their respective captions, whereas Figure 2 shows an example of graph representation of the 27 May 2020 topics. We further provide English translations of the terms composing the topics. For capitalized words and abbreviations we provide additional information in the Appendix. Table 1 shows six topics as lists of relevant terms related to 19 April 2020. The topmost topic deals with coronavirus which, as one shall expect, was a hot topic in mid-April due to the pandemic spread in Italy. The second topic deals with Walter Ricciardi, which re-tweeted an anti-Trump tweet from filmmaker Michael Moore.^6^ The third topic deals with a press release by Gabriele Gravina in which he pushed against the suspension of Italian football league competitions due to coronavirus by claiming that he does not want to be “the gravedigger of Italian football”.^7^_^8^ The fourth topic deals with the increasing number of victims due to coronavirus in Italy and the fifth one regards Lombardy, the Italian region that by far had the highest number of deaths and infected [60]. Finally, the last topic deals with Massimo Giletti, which interviewed Matteo Salvini on several COVID-19-related topics, including Walter Ricciardi’s tweet (see first topic) and possible ideas in order to relax the lockdown in Italy.^9^^6^https://www.ansa.it/sito/notizie/politica/2020/04/19/coronavirus-oms-prende-le-distanze-da-ricciardi_145af93b-ab23-4893-bf29-464a2be73821.html (in Italian).^7^https://www.goal.com/en/news/i-dont-want-be-responsible-for-the-death-of-italian-football/3rkkmzbr8fwu12mgu07l406j5^8^https://www.repubblica.it/sport/calcio/serie-a/2020/04/19/news/gravina_spera_nella_riparteza_non_sara_il_becchino_del_calcio_italiano_-254487377/ (in Italian).^9^https://www.la7.it/nonelarena/rivedila7/non-e-larena-puntata-del-19042020-20-04-2020-320316 (in Italian).

**TABLE 1:** Topics for 19/04/2020. Parameters Setup: $s=15$, $\delta=100$, $\phi=0.4$, threshold = 6

Topic	Terms	Terms (translated)
#1	virus, corona	virus, corona
#2	Ricciardi, OMS, Trump	Ricciardi, WHO, Trump
#3	calcio, italiano, studio, Gravina, becchino, fermare	football, Italian, study, Gravina, gravedigger, stop
#4	vittime, ancora	victims, more
#5	regione, Lombardia	region, Lombardy
#6	Giletti, coronavirus, nonelarena, Salvini	Giletti, coronavirus, “Non è l’arena”, Salvini

**TABLE 2:** Topics for 05/04/2020 (1). Parameters Setup: $s=8$ (or $s=15$), $\delta=100$, $\phi=0.4$, threshold = 6

Topics	Terms	Terms (translated)
#1	Gallera, Lombardia	Gallera, Lombardy
#2	Elisabetta, regina, discorso	Elizabeth, queen, speech
#3	Trump, America	Trump, (United States of) America
#4	virus, Salvini, aperte, Pasqua, corona, chiese	virus, Salvini, open, Easter, corona, churches

**TABLE 3:** Topics for 05/04/2020 (2). Parameters Setup: $s=15$, $\delta=100$, $\phi=0.25$, threshold = 6

Topics	Terms	Terms (translated)
#1	mascherina, toscano, distribuire, intelligente, obbligatorio, tutorial	safety mask, Tuscan, supply, smart, mandatory, tutorial
#2	Sky, sport	Sky, sport
#3	lega, Lombardia, governare, sanità, Fontana, Gallera	(northern) league, Lombardy, rule, health, Fontana, Gallera
#4	pensare, iniziare, fase, ISS, curva, confermare	think, start, phase, NIH, curve, confirm
#5	casa, giusto, restare, riposo, ondata, premio	home, right, stay, rest, wave, prize
#6	virus, Salvini, PD, aprire, Fiorello, chiesa	virus, Salvini, DP, open, Fiorello, church

**TABLE 4:** Topics for 05/04/2020 (3). Parameters Setup: $s=8$, $\delta=100$, $\phi=0.3$, threshold = 6

Topics	Terms	Terms (translated)
#1	mascherina, obbligo, obbligatorio, toscano, distribuire	safety mask, mandatory, Tuscan, supply
#2	restare, casa, riposo	stay, home, rest
#3	Sky, sport	Sky, sport
#4	virus, aprire, Fiorello, pregare, provare, fedele	virus, open, Fiorello, pray, try, devoted

**TABLE 5:** Topics for 16/04/2020. Parameters Setup: $s=15$ (or $s=8$), $\delta=100$, $\phi=0.4$, threshold = 6

Topics	Terms	Terms (translated)
#1	corona, virus	corona, virus
#2	morto, scrittore, Sepulveda, Luis, cileno	dead, writer, Sepulveda, Luis, Chilean
#3	Zaia, riaprire, maggio	Zaia, re-open, May
#4	disgustato, Fontana, sciacallaggio, Gallera, politico	disgusted, Fontana, slander, Gallera, politician

**TABLE 6:** Topics for 10/04/2020. Parameters Setup: $s=8$, $\delta=100$, $\phi=0.25$, threshold = 6

Topic	Terms	Terms (translated)
#1	PD, patrimoniale, solidarietà, contributo, redditi, arriva	DP, property tax, solidarity, duty, incomes, incoming
#2	Meloni, firmato, MES, Salvini, Berlusconi	Meloni, signed, ESM, Salvini, Berlusconi
#3	coronavirus, conferenza, stampa, Conte	coronavirus, press release, Conte

**TABLE 7:** Topics for 08/04/2020. Parameters Setup: $s=8$, $\delta=100$, $\phi=0.25$, threshold = 6

Topics	Terms	Terms (translated)
#1	coronavirus, Europa, Olanda, eurogruppo, solidarietà, eurobond	coronavirus, Europe, Netherlands, eurogroup, solidarity, eurobond
#2	Trump, Sanders, Biden, Bernie, primarie, ritira	Trump, Sanders, Biden, Bernie, (presidential) primary, drop off
#3	Italia, migranti, porti, quarantena, ONG, chiude	Italy, migrants, harbours, quarantine, NGO, closed
#4	oggi, guariti, covid19, ieri, casi, record	today, recovered, covid19, yesterday, cases, record

Tables 2–4 regard 5 April 2020 and we use this day in order to address the sensitivity to the cutoff parameter $\phi $ and the number $s$ of previous time windows considered in the hot terms computing. Specifically, Table 2 uses a cutoff value $\phi $ equal to 0.4 and $s$ can be either 8 or 15, leading to four topics. The first topic deals with the (rejected) motion of no confidence issued against Giulio Gallera by the Democratic Party due to the bad way (according to the Democratic Party) in which he managed the COVID-19 emergency in Lombardy.^10^ The second topic regards the hope to the nation speech by Queen Elizabeth II.^11^ The third topic, although represented by few words, may regard the briefing by Donald Trump at the White House in which he clumsily suggested hydroxychloroquine against COVID-19.^12^ The last topic, as instead, regards the (rejected) request from Matteo Salvini to let churches be open (regardless of the lockdown) for celebrating Easter.^13^ Topics in Table 3 have been obtained with cutoff value $\phi =0.25$ and $s=15$. The third topic is the same as topic #1 in Table 2, although represented by a higher number of terms. Similarly, the last topic is the same as topic #4 in Table 2 which further includes Fiorello, that replied via Instagram at Matteo Salvini’s proposal.^14^ The first topic regards the administrative order by the President of Tuscany region to make safety masks mandatory and that masks will be freely distributed door-to-door to avoid gatherings.^15^ The fourth topic cheers the news that the number of hospitalized patients starts decreasing (data from Italian National Institute of Health) and that a lockdown relaxation will be possible if the number of cases keeps decreasing.^16^ The fifth one is quite a mixed-bag, which may include the suggestion to stay at home or the tragic destiny of nursing homes in Italy.^17^ Finally, topics in Table 4 have been obtained by using a cutoff value $\phi $ equal to 0.3 and $s=8$. The first topic is the same as topic #1 in Table 3, topic #2 is likely the same as topic #5 in Table 3, topic #3 is likely the same as topic #2 in Table 3 (although this is quite hard to interpret due to very few words) and the last topic is the same as topic #4 in Table 3.^10^https://milano.repubblica.it/cronaca/2020/05/04/news/coronavirus_in_lombardia_bocciata_la_mozione_di_sfiducia_del_pd_contro_gallera_ma_ italia_viva_non_partecipa_al_voto-255685190/ (in Italian).^11^https://www.telegraph.co.uk/news/2020/04/05/queens-coronavirus-speech-full-will-succeed-better-days-will/^12^https://www.nytimes.com/2020/04/05/us/politics/trump-hydroxychloroquine-coronavirus.html^13^https://www.ansa.it/sito/notizie/politica/2020/04/05/coronavirus-salvini-permettere-le-messe-a-pasqua-_81e512ac-9a26-4ffb-8de7-c0f0ab85d763.html (in Italian).^14^https://www.ilfattoquotidiano.it/2020/04/05/coronavirus-fiorello-salvini-propone-di-aprire-le-chiese-per-pasqua-un-errore-credo-che-dio-accetti-le-preghiere-anche-di-chi-sta-a-casa/5760474/ (in Italian).^15^https://www.ansa.it/sito/notizie/cronaca/2020/04/04/coronavirus-in-lombardia-in-giro-con-le-mascherine.-anche-la-toscana-annuncia-lordinanza_6b9afb6d-1848-4366-8090-bb57ee9e1adf.html (in Italian).^16^https://www.repubblica.it/cronaca/2020/04/05/news/coronavirus_contagi_morti_guariti_bilancio_protezione_civile-253223823/ (in Italian).^17^https://bologna.repubblica.it/cronaca/2020/04/05/news/_le_case_di_riposo_sono_una_polveriera_50_morti_170_contagi-253179732/ (in Italian).

Table 5 shows four topics related to 16 April 2020. The topmost one deals with coronavirus, as expectable. The second one deals with the death due to COVID-19 of Chilean writer and journalist Luis Sepúlveda.^18^ The third topic deals with a press release by Luca Zaia, who proposed to stop the lockdown starting from 4 May 2020.^19^ The last topic (related to the previous one) regards several press releases by Attilio Fontana and Giulio Gallera regarding the COVID-19 outbreak and counter-measures in Lombardy.^20^^18^https://www.bbc.com/news/world-latin-america-52310439^19^https://www.corriere.it/politica/20_aprile_16/coronavirus-fase-2-4-maggio-anche-zaia-preme-riaprire-o-chiudere-tutto-morire-attesa-che-virus-vada-via-4c6764e2-7fdf-11ea-8804-717fbf79e066.shtml (in Italian).^20^https://milano.repubblica.it/cronaca/2020/04/16/news/coronavirus_lombardia_fontana_gallera_regione_rsa_inchieste_riapertura-254160970/ (in Italian).

Table 6 regards 10 April 2020. The first topic regards a (rejected) proposal from the Democratic Party to introduce an economic manoeuvre according to which wealthy citizens shall be waived a tax in order to support low-income people during the COVID-19 emergency.^21^ The second topic regards the (false) accusation from Giorgia Meloni and Matteo Salvini towards Giuseppe Conte of approving the European Stability Mechanism. The last topic (see also Section IV-A) regards the press release by Giuseppe Conte: in said press release, other than introducing and discussing new COVID-19-related rules and regulations, Giuseppe Conte debunked the accusation from Giorgia Meloni and Matteo Salvini (see previous topic).^22^^21^https://www.repubblica.it/politica/2020/04/10/news/il_pd_un_ contributo_di_solidarieta_da_chi_ha_un_reddito_superiore_a_ 80mila_euro_-253640966/ (in Italian).^22^https://www.corriere.it/politica/20_aprile_11/coronavirus-show-premier-conte-diretta-tv-salvini-meloni-dicono-falsita-9396558e-7b67-11ea-afc6-fad772b88c99.shtml (in Italian).

Finally, Table 7 regards 8 April 2020. The first topic regards a discussion amongst members of the European Union regarding economic manoeuvres to help European countries heavily affected by the coronavirus pandemic, with Netherlands being the most hostile member against this manoeuvre.^23^ The second topic regards Bernie Senders dropping out of the 2020 presidential race against republicans, leaving Joe Biden in charge of heading the democratic coalition.^24^ The third topic deals with an administrative order according to which Italy, due to the coronavirus pandemic, self-proclaimed as non-safe place for NGOs to dock^25^ and no migrants would be allowed on Italian soil. The last topic cheers the news that 8 April 2020 has been one of the days with few new cases and with a lot of recovered patients (more than 2000).^26^^23^https://www.ilfattoquotidiano.it/2020/04/08/coronavirus-fumata-nera-eurogruppo-stallo-su-mes-e-eurobond-olanda-noi-contro-nuova-riunione-giovedi/5763524/ (in Italian).^24^https://edition.cnn.com/2020/04/08/politics/bernie-sanders-drops-out/index.html^25^https://www.repubblica.it/cronaca/2020/04/08/news/coronavirus_sbarchi_a_lampedusa_allarme_quarantena_per_i_migranti-253444180/ (in Italian).^26^https://www.repubblica.it/cronaca/2020/04/08/news/coronavirus_bilancio_contagiati_positivi_morti_guariti_picco-253489274/ (in Italian).

## V. Conclusions

In this work we proposed an in-depth analysis of the general debate within the Italian Twitter community during the lockdown period established in Italy for security reasons due to the dramatic COVID-19 pandemic. For this purpose, it is experimented a methodological framework, grounded on a biological metaphor, able to track emerging terms and emerging topics in a given time span starting from a real-world dataset of Tweets collected during the lockdown period. The methodology served as a driver to develop a topic tracking system tailored to modern Twitter standards and specifically to the aim of retrieving buzzing terms and topics in the Italian language. The system is found capable of discovering, in an unsupervised fashion, the main emerging terms related even to socio-political events, succeeding in strongly highlighting when they are spiking, even for terms heavily and constantly used, such as, for example, the major Prime Ministers’ names. This is true also for the main related topics. The proposed system is general purpose, and can be used on streams of Twitter messages, written in any language, to detect and to track topics emerging from any socially relevant event. The topic tracking system is found sensible to some system parameters, such as the threshold for obtaining the emerging terms and the parameter for thinning the co-occurrence graph. Future works foresee the automatic search for these thresholds and an in-depth analysis of the current dataset for different granulation levels in terms of time interval length that, in the current work, is fixed in one day. Furthermore, the system will be equipped with a sentiment analysis module capable even to measure the quantity of hate speech in social media contents.

## Appendix: Glossary

**Salvini:** Matteo Salvini, Federal Secretary of the Northern League and former Deputy Prime Minister of Italy (also salvini).
**Conte:** Giuseppe Conte, current Prime Minister of Italy (also conte)
**PD:** Partito Democratico, *transl.* Democratic Party (DP), centre-left-wing Italian political party, (also pd)
**Lega:** Lega Nord, *transl.* Northern League, right-wing Italian political party (also lega)
**Calcio:** football (also calcio)
**Europa:** Europe (also europa)
**Meloni:** Giorgia Meloni, President of Fratelli d’Italia, *transl.* Brothers of Italy, a right-wing conservative Italian political party (also meloni)
**Renzi:** Matteo Renzi, former Prime Minister of Italy and Leader of Italia Viva, *transl.* Italy Alive, a centre/centre-left-wing Italian political party (also renzi)
**Borsa:** stock exchange (also borsa)
**scienza:** science
**carcere, carceri:** singular, resp. plural, form of *jail*
**CONI:** Comitato Olimpico Nazionale Italiano, *transl.* Italian National Olympic Committee (also coni)
**trump:** Donald Trump, current President of the United States of America (also Trump)
**boris:** Boris Johnson, current Prime Minister of the United Kingdom
**mes:** Meccanismo Europeo di Stabilità, *transl.* European Stability Mechanism (ESM)
**Ricciardi:** Walter Ricciardi, Italian physician, Ministry of Health collaborator and represents the Italian government to the World Health Organization executive committee during COVID-19 emergency
**OMS:** Organizzazione Mondiale della Sanità, *transl.* World Health Organization (WHO)
**Gravina:** Gabriele Gravina, President of Federazione Italiana Giuoco Calcio (FIGC), *transl.* Italian Football Federation
**Giletti:** Massimo Giletti, Italian TV presenter, host of the show *Non è l’arena* (see Table 1)
**Zaia:** Luca Zaia, President of Veneto region
**Fontana:** Attilio Fontana, President of Lombardy region
**Gallera:** Giulio Gallera, Health and Welfare Minister of Lombardy
**Sanders:** Bernie Sanders, American politician
**Biden:** Joe Biden, American politician
**ONG:** Organizzazione Non Governativa, *transl.* Non-Governmental Organizations (NGO)
**Elizabeth:** Queen Elizabeth II, Queen of the United Kingdom and Commonwealth countries
**Fiorello:** Rosario Fiorello, showman, actor, singer, TV and radio presenter
**ISS:** Istituto Superiore di Sanità, *transl.* National Institute of Health (NIH)
